# People with unbearable psychiatric suffering and a chronic death wish: Who are they and what changes do they experience in the care and expertise centre Reakiro? A Belgian cross-sectional study

**DOI:** 10.1192/j.eurpsy.2025.874

**Published:** 2025-08-26

**Authors:** T. Vanhie, S. Verdegem, L. Bemelmans, P. Onghena, J. Vandenberghe

**Affiliations:** 1 Reakiro, University Psychiatric Centre, KU Leuven; 2 KU Leuven, Leuven, Belgium

## Abstract

**Introduction:**

People with unbearable psychiatric suffering and a chronic death wish are a subgroup of patients with Severe and Persistent Mental Illness (SPMI; Moureau *et al.*, FiP 2023; 14:1094038). They suffer from at least one, but usually multiple psychiatric disorders, in a chronic course, resulting in severe limitations in psychosocial functioning (Woods *et al.*, CJP 2008; 53 725-736.). Their chronic death wish is rooted in their struggle with life and death and diminished perspective on the alleviation of their suffering. They are at risk for suicide and/or are eligible to request euthanasia as legislated in Belgium. Reakiro is a Belgian pilot project and a drop-in, care and expertise centre developing tailored mental healthcare for this target population in addition to continued care as usual.

**Objectives:**

To describe psychological and existential characteristics, care needs, experienced changes and the correlations between these variables in 107 consecutive Reakiro patients who agreed to participate in this study.

**Methods:**

The Beck Scale for Suicide Ideation (BSSI), Dutch Empowerment Scale (DES), Herth Hope Index (HHI), Meaning In Life Measure (MILM), Existential Concerns Questionnaire (ECQ), Outcome Questionnaire-45 (OQ-45) and the Change Questionnaire (CQ) were administered combined with an assessment of their life and death wish and of how they experience the care in Reakiro.

**Results:**

The BSSI (M = 21.78, SD = 8.48), DES (M = 113.39, SD = 20.42), HHI (M = 24.15, SD = 5.40), MILM (M = 5.39, SD = 1.50), ECQ (M = 62.13, SD = 12.10) and OQ-45 (M = 98.93, SD = 20.50) show heightened levels of suicidality, existential anxiety and symptomatology and lowered levels of hope, meaning in life and empowerment, compared to other psychiatric and non-clinical samples. Longer trajectories in Reakiro were correlated with heightened hope and lowered symptomatology, but not with suicidality. The preliminary CQ-analysis revealed 31 participants reporting positive changes in their relation to life and death, to self and others and to hope and future; 8 participants reported negative changes in their relation to life and death.

**Conclusions:**

The results depict a concrete profile of the severity of the suffering and suicidality in these patients. The mixed-methods design reveals a major group that does not report any change and suffers greatly, but also a minor group that reports positive changes and even lowered suicidality. Clinical implications for psychiatric healthcare professionals will be discussed.

**Disclosure of Interest:**

None Declared

